# Cardiorenal syndrome followed by acute hepatitis C in a patient with acute myeloid leukemia

**DOI:** 10.12669/pjms.303.4376

**Published:** 2014

**Authors:** Romeo-Gabriel Mihaila

**Affiliations:** 1 Romeo-Gabriel Mihaila, MD, PhD, "Lucian Blaga" University of Sibiu, Faculty of Medicine, Str Lucian Blaga, nr 2A, Cod 550169, Sibiu, Romania.

**Keywords:** Acute leukemia, Acute hepatitis, Cardiorenal syndrome, Cholesterol, Hemodialysis

## Abstract

Cardiorenal syndrome involves altering cardiac and renal function. These patients frequently develop resistance to diuretic therapy, so that ultrafiltration should be applied in emergency for saving them. Concomitant presence of an active hematologic malignancy represents an important complicating factor. We present the case of an elderly patient with acute myeloid leukemia, appeared on the background of myelodysplastic syndrome who, during marrow aplasia occurred after the first course of induction chemotherapy, developed a cardiorenal syndrome, which required repeated sessions of hemodialysis. Complete hematologic remission and efficiency of fluid depletion therapy allowed the second course of polychemotherapy, after which the patient developed an acute hepatitis C. After 8 months of complete hematologic remission that persists, the patient will be put on the standard antivirusologic treatment.

## INTRODUCTION

Patients with acute leukemia often have underlying conditions which may decompensate or cause complications that may endanger life. The situation is even worse if these decompensations or complications arise when acute leukemia is not in complete remission. In addition, acute leukemia predispose to the occurrence of complications and iatrogenic disorders. If the majority of the complications creates difficulties in acute leukemia therapy, the relationship between acute leukemia and some infections - such as hepatitis C virus (HCV) infection - is questionable. Sometime long remissions of acute leukemia were observed, in patients who had been infected with HCV. In addition to nonspecific stimulation of the immune system of the patient, there may be another explanation, too: leukemia cells use cholesterol for their own proliferation and during HCV infection cholesterol synthesis decreases, including the cholesterol available for them.

## CASE PRESENTATION

The patient, aged 68, known with gastro-oesophageal reflux disease and chronic asthma, was admitted to the hematology service for bone pain, skin pallor and petechiae on the legs. In addition, he presented with fatigue, epigastric pain, heartburn, nausea, loss of appetite, occipital headache and vertigo - clinical manifestations that occurred a month ago. He had normal weight, was afebrile, with no pathological changes detected on physical examination of the respiratory and cardiovascular systems. He was discrete sensitive to palpation in the epigastric region and the liver was slightly increased in volume (13 cm on the medioclavicular line) and had slightly higher consistency.

Biological samples showed pancytopenia, positive inflammatory tests, slight decrease in serum cholinesterase and albumin; there were normal: creatinine, transaminases, immunoelectrophoresis and HBs antigen, and anti-HCV antibodies were absent. Myelogram showed a trilinear dysplasia and a blasts percentage of 25%, of which 5% were peroxidazo-positive. Flowcytometry from peripheral blood isolated a blasts population of 30% with the following phenotype: CD34+, CD33+, HLA-DR+, CD41-, CD61-. CD64-, CD3i.c.-, CD5-, CD10-, CD14-, CD19-, CD20-, CD22-, CD235-, TdT-, MPXi.c.+ (6%). Molecular biology examination did not detect mutations with poor prognosis. Ultrasonographically, the liver was diffusely hyperechogenic, with no signs of portal hypertension; the long axis of the spleen measure 10 cm. Imagistically, he had a widened peribronhovascular interstitium para- and infrahilar and radiological signs of left coxarthrosis. Dental consultation discovered a periodontal abscess (4.8) and echocardiography measured an ejection fraction of 53%.

This acute myeloid leukemia that occurred on the background of a myelodysplastic syndrome was treated with cytarabine 250 mg/day, 7 days + idarubicin 15 mg/day, 3 days. Simultaneously, we treated: the periodontal abscess (with vancomycin, imipenem and metronidazole) and the gastroesophageal reflux disease, and performed the prophylaxis of asthma exacerbations. Intestinal decontamination was performed during aplasia and he was transfused with red cell and platelet concentrates. During aplasia, clinical condition began to worsen progressively, and low grade fever (37.8°C) appeared; he had cough with purulent sputum in small quantities, wheezing, heartburn and decreased urine output to 1000 mL/24h. On physical examination he presented with decreased vesicular murmur and diffuse wheezing and crepitation who ascended to bilateral subclavian region; blood pressure reached 160/90 mmHg and heart rate was 96/minut; he developed leg edema, which then expanded and progressed to the stage of anasarca, while creatinine increased, but not more than 3.39 mg/dL, and blood urea increased to 147 mg/dL.

Radiologically, he had signs of acute pulmonary preedema (imprecisely demarcated opacities with trend confluence, located mostly in perihilar region) and small bilateral pleural fluid collections. Echocardiography showed a left ventricular ejection fraction of 85%, and the presence of significant pulmonary hypertension. Since being treated with furosemide diuresis decreased to 400 mL/day, it was decided to perform hemodialysis in intensive care monitoring service. It was showed that urine culture was positive with Enterobacter, sensitive to meropenem, which was treated with. After 10 hemodialysis sessions and diuretic treatment edema decreased significantly and diuresis reached 1800 mL/day; respiratory and cardiac symptoms also improved. Myelogram control made at the output of aplasia noted that acute leukemia was in complete remission. He was released and returned after a month, with pallor and swelling that had also a hypoproteinemic component. Edema disappeared, and serum creatinine decreased from 2.93 mg/dL to 1.57 mg/dL after diuretic treatment, albumin, plasma and 2 more hemodialysis sessions,. He received a second course of polychemotherapy (cytarabine 200 mg/day, 7 days + idarubicin 10 mg/day, 3 days). During aplasia he had an episode of fever without bacteriological documentation, submitted under antibiotherapy, and was transfused with red cell and platelet concentrates.

After a month, he returned asthenic, with loss of appetite, and chemotherapy could not be performed because of hepatic cytolysis (AST 372 IU/L, ALT 864 IU/L). HBs antigen and anti-HCV antibodies were negative again. After a month, he had: AST 843 IU/L, ALT 1948 IU/L, total bilirubin 2.26 mg/dl, and direct bilirubin 1.56 mg/dL. Virological determinations were performed this time: IgM anti-hepatitis A virus antibodies (negative), IgM anti-cytomegalovirus antibodies (absent), hepatitis B virus DNA (undetectable), hepatitis C virus RNA - positive (1,371,430.00 IU/mL). After another month he had: normal AST, ALT 50 IU/L, creatinine 1.26 mg/dL, cholesterol 209 mg/dL, and anti-hepatitis C virus antibodies were positive. Myelogram established that acute leukemia is still in complete remission. FibroTest had a value of 0.62 (score F3 - portal and periportal fibrosis with numerous septa) and ActiTest - 0.97 (score A3 – severe necro-inflammatory activity – portal inflammatory injuries and hepatocyte necrosis). After another two months, acute leukemia was still in complete remission, and hepatic cytolysis - minimal. The patient will begin treatment with pegylated interferon + ribavirin.

## DISCUSSION

The elderly patient had acute leukemia on the background of a myelodysplastic syndrome (trilinear dysplasia). His performance status was good at first, but it deteriorated during the first post-chemotherapy aplasia after the emergence of acute bronchitis, on the background of chronic asthma. This inflammatory process contributed at increasing the resistance in pulmonary arterial circulation and the pulmonary hypertension with right heart decompensation. Although left ventricular ejection fraction was normal, increased retrograde venous pressure, including at the renal veins, with a diminishing of transrenal perfusion pressure, contributed to decreasing the glomerular filtration rate^1^, hydro-saline retention emergence, refractory to diuretic therapy, which required hemodialysis. A cardiorenal syndrome can also appear in patients with normal left ventricular ejection fraction. This can be explained by the involvment of neurohormonal factors, the disruption of intrarenal hemodynamics, and the decreasing of transrenal perfusion pressure.^1^ In our opinion, this cardiorenal syndrome fits best in type I, although we cannot exclude type II and V. It is known that in addition to the five known types there are also combined forms in which our patient can be included because he had chronic asthma and chronic pulmonary heart, initially compensated, that have progressed to decompensation in the context of acute respiratory infection. The daily 160 mg of furosemide have proven to be ineffective. We have not increased the dose because a high dose of diuretics could contribute to neurohormonal activation and vasoconstriction which are involved in worsening renal function.^1^^,^^2^ Extracorporeal ultrafiltration, realised during hemodialysis, is an efficient way to remove excess body fluid in patients with chronic heart failure refractory to medical treatment.^3^ Hemodialysis and ultrafiltration are also indicated for the treatment of acute cardiorenal syndrome^4^, as those of our patient. The patients with severe heart failure have the best chance of benefiting from this method of fluid removal.^5^ Unfortunately, the optimal rate to fluid removal was not established.^3^

The access for acute dialysis increases the infection risk^6^, that adds those related to transfusions made. So our patients developed an acute hepatits C on the background of pre-existing liver fibrosis favored by previous right heart decompensation. Infection with hepatitis viruses is relatively common among patients with hematologic malignancies. Thus, in Russia, in a group of 257 patients, of whom 205 had acute leukemia, only 29.4% had no specific markers of infection with hepatitis viruses, and 81.4% of those with hepatitis C had markers of coinfection with hepatitis B, too.^7^ The presence of hepatitis C virus infection to the presented patient can cause difficulties regarding further chemotherapy in case of acute leukemia relapse. Therefore, we opted for therapy with pegylated interferon + ribavirin, although he has not yet developed chronic hepatitis. On the other hand, the period of complete remission of acute leukemia (over 8 months) after only two courses of induction chemotherapy (without high-dose cytarabine, due to impaired performance status after the first treatment) is surprisingly long. The explanation may be low cholesterol synthesis during infection with hepatitis C^8^ and high cholesterol needs of the leukemia cells, which they use for their own proliferation.^9^

We expect that during the antiviral therapy the patient will be predisposed to the occurrence of malignant hemopathy relapse by increasing cholesterol available for leukemic cells. Therefore, the patient has indication for therapy with some statins, which lower cholesterol synthesis and have other useful pleiotropic effects in both acute leukemia and in antiviral therapy - reduce HCV replication, especially in combination with pegylated interferon + ribavirin, and significantly increase the likelihood of sustained complete virological response, as observed in clinical studies with fluvastatin.^10^
